# Polymorphisms of melatonin receptor genes and their associations with egg production traits in Shaoxing duck

**DOI:** 10.5713/ajas.17.0828

**Published:** 2018-04-12

**Authors:** Peishi Feng, Wanqiu Zhao, Qiang Xie, Tao Zeng, Lizhi Lu, Lin Yang

**Affiliations:** 1 College of Animal Science, South China Agricultural University, Guangzhou 510642, China; 2 Institute of Animal Husbandry and Veterinary Medicine, Zhejiang Academy of Agricultural Sciences, Hangzhou 310021, China; 3 College of Animal Science, Zhejiang University, Hangzhou 310058, China

**Keywords:** *MTNR1A*, *MTNR1B*, *MTNR1C*, Single Nucleotide Polymorphism Polymorphism, Egg Production Traits

## Abstract

**Objective:**

In birds, three types of melatonin receptors (*MTNR1A*, *MTNR1B*, and *MTNR1C*) have been cloned. Previous researches have showed that three melatonin receptors played an essential role in reproduction and ovarian physiology. However, the association of polymorphisms of the three receptors with duck reproduction traits and egg quality traits is still unknown. In this test, we chose *MTNR1A*, *MTNR1B*, and *MTNR1C* as candidate genes to detect novel sequence polymorphism and analyze their association with egg production traits in Shaoxing duck, and detected their mRNA expression level in ovaries.

**Methods:**

In this study, a total of 785 duck blood samples were collected to investigate the association of melatonin receptor genes with egg production traits and egg quality traits using a direct sequencing method. And 6 ducks representing two groups (3 of each) according to the age at first eggs (at 128 days of age or after 150 days of age) were carefully selected for quantitative real-time polymerase chain reaction.

**Results:**

Seven novel polymorphisms (*MTNR1A*: g. 268C>T, *MTNR1B*: g. 41C>T, and g. 161T>C, *MTNR1C*: g. 10C>T, g. 24A>G, g. 108C>T, g. 363 T>C) were detected. The single nucleotide polymorphism (SNP) of *MTNR1A* (g. 268C>T) was significantly linked with the age at first egg (p<0.05). And a statistically significant association (p<0.05) was found between *MTNR1C* g.108 C>T and egg production traits: total egg numbers at 34 weeks old of age and age at first egg. In addition, the mRNA expression level of *MTNR1A* in ovary was significantly higher in late-mature group than in early-mature group, while *MTNR1C* showed a contrary tendency (p<0.05).

**Conclusion:**

These results suggest that identified SNPs in *MTNR1A* and *MTNR1C* may influence the age at first egg and could be considered as the candidate molecular marker for identify early maturely traits in duck selection and improvement.

## INTRODUCTION

Melatonin (N-acetyl-5-methoxytryptamine) is an important hormone that is synthesized mainly in the pineal gland, and has a profound effect on serval physiology process including circadian rhythm and reproduction through its special receptors in birds [1–3]. Three melatonin receptor subtypes, *MTNR1A* (alias *MT1*, *Mel1a*), *MTNR1B* (alias *MT2*, *Mel1b*), and *MTNR1C* (alias *Mel1c*), which belong to the superfamily of G protein-coupled receptors [4], have been cloned in birds [5,6].

In chicken, melatonin receptor subtypes were identified in ovaries [7,8], suggesting that melatonin directly affects ovarian function through activating of multiple receptors. In geese, the expression levels of *MTNR1A*, *MTNR1B*, and *MTNR1C* initially increased and later decreased during follicular development cycle, indicating that melatonin receptors participated in activating small white follicles and small yellow follicles to develop into subsequent greater hierarchical follicles [9]. In addition, *in situ* hybridization of *MTNR1C* mRNA combined with immunocytochemistry for gonadotropin-inhibitory hormone (GnIH), a key neurohormone controlling avian reproduction by inhibiting gonadal development [10], revealed a clear cellular colocalization of *MTNR1C* mRNA and GnIH in paraventricular nuclei [11]. It seems that melatonin most likely acts directly on GnIH neurons through *MTNR1C* to induce *GnIH* expression and regulate avian reproduction.

Shaoxing duck is a Chinese dominant layer breed, characterized by small body size, early maturity and high productivity. Modern Shaoxing duck achieve 50% egg production by 140 days of age, and the mean number of eggs at 500 days old was more than 300 [12]. Shaoxing ducks have been considered as a good source of duck eggs in China, and improving its egg production performance is of primary interest to breeders and farmers. The identification of single nucleotide polymorphisms (SNP) in candidate genes associating with economically important traits has become a powerful tool for genetic improvement of animal selection and production.

Numerous studies have investigated the relationship between melatonin receptor subtypes and egg production traits in different species, and made melatonin receptor genes potential candidate genes for QTLs [13,14]. However, whether or not nucleotide polymorphisms of *MTNR1A*, *MTNR1B*, and *MTNR1C* are associated with the egg production traits and egg quality traits in ducks is still unknown. Therefore, this experiment aimed to detect the SNPs in *MTNR1A*, *MTNR1B*, and *MTNR1C* and explore their associations with egg production traits and egg quality traits. The expression levels of these genes in ovary of ducks at different age at first egg were also determined.

## MATERIALS AND METHODS

### Performance traits and tissue collection

A total of 785 female Shaoxing ducks were randomly selected and raised in separate cages under similar environmental conditions and diet. The egg production traits of all ducks were recorded throughout the egg production process in terms of age at first egg, egg weight at 34 weeks old and total number of eggs at 34 weeks old and 72 weeks old. Egg collection for egg quality measurements took place at 34 weeks old. The following traits were recorded: egg shape index, shell thickness, shell strength, albumen height, Haugh unit score and eggshell color.

The blood samples of all the 785 individual ducks were collected from the wing vein using vacuum tubes containing dipotassium ethylene diamine tetraacetate (EDTA-K_2_) as an anticoagulant for further SNP analysis. To investigate the expression of the multiple forms of melatonin receptor mRNA, 6 ducks representing two groups (3 of each) according to the age at first egg (at 128 days of age or after 150 days of age) were carefully selected at age of 150 days. Ovaries were sampled after slaughter. These experiments were conducted in accordance with the Law of the People’s Republic of China on Animal Protection.

### SNP discovery and genotyping

Based on the complete DNA sequence of *Anas platyrhynchos* genomic DNA sequence (NCBI accession no. NW_004676748.1), four pairs of primers were designed to amplify the target regions for SNP genotyping using the Primer Premier 6.0 software. Primer sequences are listed in Table 1.

Polymerase chain reaction (PCR) was carried out in a total volume of 15 μL consisting of 1 μL genomic DNA, 0.15 μL (10 μM) of each primer, 1.5 μL 10× buffer, 1.5 μL (25 mmol/L) MgCl_2_, 0.3 μL (10 mmol/L) dNTPs, and 1.5 U Taq DNA polymerase. PCR conditions were as follow: 95°C for 3 min, followed by 35 cycles of 95°C for 15 s, 55°C for 15 s, 72°C for 30 s, and a final extension of 72°C for 10 min. The PCR products were sequenced commercially using ABI 3730XL automated sequencer (Applied Biosystems, Carlsbad, CA, USA) after being purified and extended. SNPs were identified by looking for multiple peaks at the same base pair.

### Statistical analysis

The genotypic frequencies were calculated for each individual and the Hardy-Weinberg equilibrium was analyzed using the Chi-square test of PopGene Version 1.32. Pairwise tests for linkage disequilibrium (LD) were performed for each SNP using the SHEsisPlus online software platform (http://shesisplus.bio-x.cn/SHEsis.html). The egg production traits were compared among the genotypes. The association between the SNPs and different traits in 785 ducks were analyzed using SPSS 22 with the model *Y* = *μ*+*G*+*L*+*G*×*L*+*e*, where Y is the dependent variable (analyzed traits), *μ* is the overall mean, *G* is the genotype with a variation for the candidate gene, *L* is the fixed effect of breed, *G*×*L* is the interaction between the genotype and duck population (a fixed effect), and *e* is the random error. The significance of the least squares means was tested with lest significance difference test.

### Quantitative real-time polymerase chain reaction

Total RNA was extracted from ovary samples using Trizol reagent according to the manufacturer’s protocol. cDNA was synthesized from the total RNA according to the manufacturer’s protocol of TransScript First-Strand cDNA Synthesis SuperMix (TransGen, Beijing, China). Quantitative real-time PCR was performed using ABI 7500 (Applied Biosystems, Foster City, CA, USA). The mRNA expression levels were determined by Applied Biosystems real-time PCR using the *MTNR1A*, *MTNR1B*, and *MTNR1C* mRNA-specific primers and the *β-acting* gene as the internal control (Table 1). The 20 μL amplification reaction contained 10 μL of SYBR Green Universal PCR Master Mix, 2 μL cDNA, 0.4 μM of each primer and nuclease free water up to 20 μL. Thermal parameter used to amplify the template started with an initial denaturation at 94°C for 3 min followed by 40 cycles of 94°C for 10 s and annealing at 60°C for 30 s. The relative expression levels of the genes test were calculated using the 2^−ΔCt^ method. Groups with different letters were significantly different at p<0.05.

## RESULTS

### Genetic polymorphism of melatonin receptor genes

**Figure 1:**
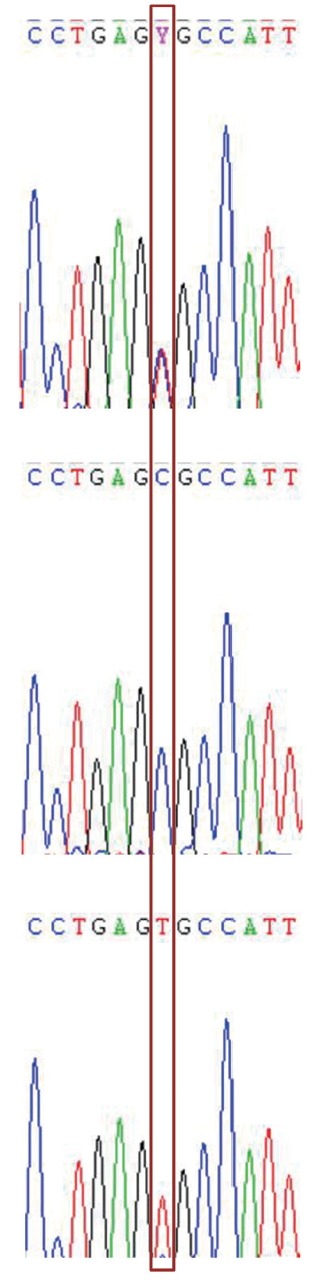
The C/T transition at base position 108 in the exon of *MTNR1C* gene. The box shows the single nucleotide polymorphism location. *MTNR1C*, melatonin receptor.

Seven SNPs were identified in all three melatonin receptor gene exons respectively by direct sequencing (Figure 1), their corresponding allele and genotype frequencies are presented in Table 2. Hardy-Weinberg equilibrium tests showed that the alleles of *MTNR1A* g. 268C>T, *MTNR1C* g. 10C>T, and *MTNR1C* g. 108C>T were in Hardy-Weinberg equilibrium (p>0.05), and others deviated from the Hardy-Weinberg equilibrium (p<0.05) (Table 1). The LD tests of Shaoxing duck population showed that two SNPs (g. 41C>T, g. 161T>C) identified in *MTNR1B* gene were completely linked (D′ = 1.0 and r^2^ = 1.0) (Supplementary Table S1), providing only three possible haplotypes.

### Association of SNPs with egg production traits and egg quality traits

The association between the SNPs and egg production traits are shown in Table 3. For the *MTNR1A* g. 268C>T locus, age at first egg of the genotype CT was significantly less than that of the genotype TT (p<0.05). The g. 108C>T locus of *MTNR1C* was significantly associated with age at first egg and total number of eggs during 34-week egg-laying period (p<0.05). For *MTNR1C* g. 108C> T, the egg number of the genotype CT at 34 weeks old was significantly higher than that of the genotype CC (p<0.05), and ducks with the genotype TT exhibited significantly earlier age at first egg than that of the genotype CC (p<0.05). No significant association was found among other SNPs and egg weight, total number of eggs at 34 weeks old, total number of eggs at 72 weeks old or age at first egg (p>0.05).

As shown in Table 4, there were no significant association of 7 SNPs with egg quality trait. Furthermore, relation of SNPs with the color of eggshell was analyzed using chi-square test. When white eggshell was compared with blue eggshell, no significant differences in genotypes of *MTNR1B* or *MTNR1C* were observed except *MTNR1A* (p>0.05). The genotype TT and CT of g. 268C>T in *MTNR1A* were more common in Shaoxing ducks laying white eggshell compared with blue eggshell (Supplementary Table S2), suggesting *MTNR1A* g. 268C>T SNP may affect the eggshell color.

### Expression of melatonin receptor mRNA in ovary

**Figure 2:**
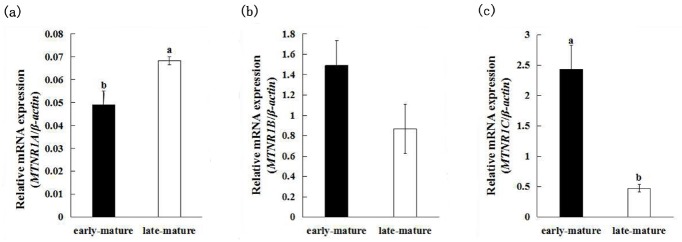
Results of expression of melatonin receptor subtypes mRNA in ovaries between early-mature group and late-mature group. (a) Comparison of mRNA expression of *MTNR1A* in ovaries between early-mature group and late-mature group. (b) Comparison of mRNA expression of *MTNR1B* in ovaries between early-mature group and late-mature group. (c) Comparison of mRNA expression of *MTNR1C* in ovaries between early-mature group and late-mature group. *MTNR1A*, *MTNR1B*, *MTNR1C*, melatonin receptor.

The expression of *MTNR1A*, *MTNR1B*, and *MTNR1C* between early-mature (first egg at 128 days of age) group and late-mature (first egg after 150 days of age) group showed different patterns (Figure 1). The late-mature group had significantly higher *MTNR1A* mRNA expression (0.0684±0.0017) compared to the early-mature group (0.0491±0.0060) (p<0.05) (Figure 2a), whereas there was no significant difference of *MTNR1B* mRNA expression between the early-mature group (1.491±0.4580) and the late-mature group (0.869±0.2435) (p> 0.05) (Figure 2b). The *MTNR1C* expression level of ovary was significantly higher in the early-mature group (2.4328±0.3936) than in late-mature group (p<0.05) (0.4756±0.0595) (Figure 2c).

## DISCUSSION

Age at first egg, an important trait indicating sexual maturation and egg production performance, was negatively correlated with number of eggs [15–17]. However, age at first egg was controlled by polygenes [17] with low to moderate estimated heritability ranging from 0.13 to 0.20 [18–20], making the conventional breeding method ineffective. Therefore, molecular assisted selection becomes a powerful tool for improving egg production traits and raising economic benefits.

As previously reported in other avian species, melatonin regulates gonadal maturation by suppressing luteinizing hormone (LH) secretion, and stimulating GnIH and gonadotropin releasing hormone secretion [21–24]. *MTNR1C* mRNA colocalized with GnIH neurous [11] indicating that melatonin participates in gonadal maturation through binding its receptors. Therefore, melatonin receptors would be possible markers for selecting an early maturing breed. Although there were many studies seeking correlations between markers of candidate genes and age at first egg, such as growth hormone, prolactin, neuropeptide Y, follicle-stimulating hormone receptor, and SH3-domain GRB2-like 2 [25–29], little was known regarding polymorphisms in melatonin receptors of duck.

Our study demonstrated that g. 268C>T of *MTNR1A* gene shows a strong association with age of first egg. Beside, g. 268C>T of *MTNR1C* gene was found to be associated with total number of eggs at age of 34 weeks and age at first egg. A study in chickens also showed that SNPs (JQ249890:g. 384T>C, JQ249891:g. 387 T>C) locating at *MTNR1A* and SNP (JQ249896:g. 294 G>A) locating at *MTNR1C* were significantly linked with age at first egg [14]. The novel SNPs which was founded in our study, indicated that melatonin receptor genes may affect age at first egg and involve in sexual mature of ducks.

The exact role of melatonin receptors in different species during the reproductive process is not well understood. It was reported that melatonin receptor density showed a striking downregulation in brain when songbirds were less than fully reproductively mature, and subsequent resumed during photorefractory state [30]. While Abd et al [31] reported that *MTNR1B* and *MTNR1C* expression increased accompanying a delay in sexual maturity of Japanese quails. In our study, the expression of *MTNR1C* mRNA in late-mature group was significantly decreased compared to the early-mature group. Summarizing the results of the above researches, it is reasonable to suggest that *MTNR1C* activates avian sexual mature by down-regulating its expression level. The *MTNR1A* mRNA expression in our experiment showed a contrary tendency with *MTNR1C* mRNA, suggesting melatonin receptors regulate ovarian function through different mechanisms. Further studies are continuing to elucidate the exact role of melatonin receptors in avian sexual maturity.

Collectively, the results of present study suggest that the *MTNR1A* and *MTNR1C* genes play an important role in egg production of ducks, but also SNPs in these genes could be used as markers in molecular marker-assisted selection for duck reproduction traits.

## Supplementary Data



## Figures and Tables

**Table 1:** Primers and sequencing information

Gene	Primer	Primer sequences (5′→3′)	Product size (bp)
Genotyping			
*MTNR1A* exon 2	P1	F: CAACGGATGGAACCTGGGATA R: TGGGCACGATAGCAACAACT	216
*MTNR1B* exon 1	P2	F: TAGGTAACGCATTTGTGGTG R: AATACCCAGACTAAGGAGAC	282
*MTNR1C* exon	P3	F: AGATGGGAGGCAGAGTGAAG R: TGTAGCAGTAGCGGTTGATT	321
*MTNR1C* exon	P4	F: ACTTCTTCGTTGGGTCCTTG R: TGTTATTGTTCGTTACTGCTG	548
Gene expression			
*MTNR1A*	Q1	F: TAGTGGCTTCTTGATGGG R: AACAGGTTGGGCACGATA	186
*MTNR1B*	Q2	F: GTGTATAGCTGCTGGAAC R: CACAACAGTGATAGGGAC	198
*MTNR1C*	Q3	F: ATCGCAATCAACCGCTAC R: CAAGGACCCAACGAAGAA	144
*β-acting*	Q4	F: GCTATGTCGCCCTGGATTTC R: CACAGGACTCCATACCCAAGAA	168

*MTNR1A*, *MTNR1B*, *MTNR1C*, melatonin receptor.

**Table 2:** Genotypic and allelic frequency at the SNP loci of *MTNR1A*, *MTNR1B*, and *MTNR1C* genes in the Shaoxing duck population

SNP	Genotype	No. of ducks	Genotype frequency	Allele	Allele frequency	χ^2^
g. 268C>T	CC	461	0.587	C	0.767	0.013
(*MTNR1A*)	CT	282	0.359	T	0.233	
	TT	42	0.054			
g. 41C>T	CC	298	0.380	C	0.581	23.742*
(*MTNR1B*)	CT	316	0.403	T	0.419	
	TT	171	0.218			
g. 161T>C	TT	298	0.380	T	0.581	23.742*
(*MTNR1B*)	TC	316	0.403	C	0.419	
	CC	171	0.218			
g. 10C>T	CC	252	0.321	C	0.563	0.223
(*MTNR1C*)	CT	380	0.484	T	0.437	
	TT	153	0.195			
g. 24A>G	AA	167	0.213	A	0.486	6.821*
(*MTNR1C*)	AG	429	0.546	G	0.514	
	GG	189	0.241			
g. 108C>T	CC	499	0.636	C	0.795	0.450
(*MTNR1C*)	CT	250	0.318	T	0.205	
	TT	36	0.046			
g. 363T>C	TT	339	0.432	T	0.643	4.907*
(*MTNR1C*)	TC	332	0.423	C	0.357	
	CC	114	0.145			

SNP, single nucleotide polymorphism; *MTNR1A*, *MTNR1B*, *MTNR1C*, melatonin receptor.

* p<0.05 was accepted to be statistically significant when the data were analyzed using a Pearson’s goodness-of-fit chi-square test (degree of freedom = 1).

**Table 3:** Association between polymorphisms in *MTNR1A*, *MTNR1B*, and *MTNR1C* genes and egg production traits in Shaoxing ducks1)

SNP	Genotype	Association of SNP with egg production traits			

		Egg weight	Number of eggs at 34 weeks of age	Number of eggs at 72 weeks of age	Age at first egg
g. 268C>T	CC	70.45±5.17	75.99±5.00	303.23±12.93	146.51±15.18^ab^
(MTNR1A)	CT	69.61±5.56	77.49±4.94	302.64±11.43	143.74±15.10^b^
	TT	68.57±8.20	74.95±3.73	298.19±15.49	146.55±15.30^a^
g. 41C>T	CC	70.13±5.46	76.64±4.87	303.84±15.23	146.21±15.77
(MTNR1B)	CT	70.33±5.59	75.88±4.98	304.28±16.49	145.85±14.87
	TT	69.37±5.52	77.29±4.92	299.23±11.94	143.68±14.73
g. 161T>C	TT	70.13±5.46	76.64±4.87	303.84±15.23	146.21±15.77
(MTNR1B)	TC	70.33±5.59	75.88±4.98	304.28±16.49	145.85±14.87
	CC	69.37±5.52	77.29±4.92	299.23±11.94	143.68±14.73
g. 10C>T	CC	69.98±5.59	75.53±4.81	303.49±13.99	145.95±14.44
(MTNR1C)	CT	69.92±5.57	77.23±4.79	306.12±14.44	145.26±15.73
	TT	70.46±5.34	76.16±5.40	306.40±16.93	145.45±15.15
g. 24A>G	AA	70.44±5.28	78.25±4.10	302.93±14.29	144.54±13.60
(MTNR1C)	AG	69.85±5.63	76.11±4.86	296.98±13.30	145.62±15.41
	GG	70.13±5.51	75.72±5.68	304.29±17.11	146.16±16.06
g. 108C>T	CC	70.25±5.73	75.09±5.34^b^	298.91±11.83	146.50±15.75^a^
(MTNR1C)	CT	69.79±5.24	78.91±3.76^a^	302.65±14.23	144.28±14.28^ab^
	TT	68.98±4.56	78.78±4.56^ab^	314.21±15.37	140.56±12.03^b^
g. 363T>C	TT	69.68±5.47	76.16±4.83	301.38±12.82	145.73±14.54
(MTNR1C)	TC	70.43±5.53	76.53±4.82	303.74±14.12	145.68±15.67
	CC	70.01±5.68	77.23±5.56	298.16±10.73	144.41±15.81

*MTNR1A, MTNR1B, MTNR1C*, melatonin receptor; SNP, single nucleotide polymorphism; SD, standard deviation.

1) Data are expressed as mean±SD.

ab Values followed by different alphabets differ significantly (p<0.05).

**Table 4:** Association between polymorphisms in *MTNR1A*, *MTNR1B*, and *MTNR1C* genes and egg quality traits in Shaoxing ducks1)

SNP	Genotype	Association of SNP with egg quality traits					

		Egg shape index	Shell thickness (mm)	Shell strength (kg/cm^2^)	Yolk color	Albumen height (mm)	Haugh unit (HU)
g. 268C>T	CC	1.32±0.04	0.45±0.04	4.82±0.71	11.63±0.24	6.74±1.21	76.23±8.93
(MTNR1A)	CT	1.31±0.06	0.43±0.03	4.78±0.68	11.68±0.31	6.81±1.82	76.73±8.49
	TT	1.33±0.03	0.45±0.06	4.73±0.53	11.48±0.73	6.73±1.63	75.19±8.03
g. 41C>T	CC	1.34±0.03	0.44±0.03	4.81±0.73	11.59±0.51	6.83±1.35	75.87±7.81
(MTNR1B)	CT	1.33±0.06	0.45±0.06	4.69±0.64	11.50±0.39	6.74±1.23	74.87±8.92
	TT	1.34±0.06	0.44±0.05	4.77±0.76	11.41±0.18	6.71±1.83	75.27±7.38
g. 161T>C	TT	1.34±0.03	0.44±0.03	4.81±0.73	11.59±0.51	6.83±1.35	75.87±7.81
(MTNR1B)	TC	1.33±0.06	0.45±0.06	4.69±0.64	11.50±0.39	6.74±1.23	74.87±8.92
	CC	1.34±0.06	0.44±0.05	4.77±0.76	11.41±0.18	6.71±1.83	75.27±7.38
g. 10C>T	CC	1.33±0.03	0.45±0.06	4.68±0.62	11.63±0.71	6.68±1.64	75.29±8.06
(MTNR1C)	CT	1.34±0.05	0.46±0.04	4.78±0.68	11.64±0.78	6.76±1.57	76.64±6.83
	TT	1.32±0.06	0.45±0.03	4.79±0.81	11.67±0.77	6.75±1.53	76.47±8.18
g. 24A>G	AA	1.31±0.05	0.43±0.02	4.84±0.69	11.61±0.62	6.74±1.62	75.85±8.04
(MTNR1C)	AG	1.32±0.06	0.44±0.04	4.83±0.72	11.59±0.64	6.72±1.63	77.78±7.73
	GG	1.31±0.08	0.43±0.03	4.79±0.93	11.62±0.59	6.75±1.60	76.84±8.90
g. 108C>T	CC	1.33±0.06	0.44±0.03	4.73±0.99	11.72±0.37	6.69±1.46	76.23±8.93
(MTNR1C)	CT	1.32±0.05	0.45±0.02	4.78±0.63	11.66±0.84	6.71±1.62	74.81±6.29
	TT	1.31±0.07	0.44±0.05	4.68±0.61	11.65±0.52	6.70±1.48	76.05±7.84
g. 363T>C	TT	1.33±0.07	0.43±0.05	4.69±0.73	11.68±0.63	6.72±1.27	75.87±7.82
(MTNR1C)	TC	1.32±0.05	0.44±0.03	4.70±0.74	11.73±0.71	6.75±1.53	76.83±8.03
	CC	1.33±0.06	0.44±0.05	4.74±0.85	11.59±0.64	6.71±1.83	77.81±7.82

*MTNR1A*, *MTNR1B*, *MTNR1C*, melatonin receptor; SNP, single nucleotide polymorphism; SD, standard deviation.

1) Data are expressed as mean±SD.
