# Supplementary material for: Polymorphisms of melatonin receptor genes and their associations with egg production traits in Shaoxing duck
Source: Asian-Australas J Anim Sci. 2018 Apr 12;31(10):1535–41. doi: 10.5713/ajas.17.0828 (PMC6127595; doi:10.5713/ajas.17.0828)
Supplement: Supplementary file 1 [file ajas-31-10-1535-supplementary.pdf]

Table S1 Linkage disequilibrium tests of 7 loci in duck *MTNR1A*, *MTNR1B* and *MTNR1C* genes*

	g. 268C>T (*MTNR1A*)	g. 41C>T (*MTNR1B*)	g. 161T>C (*MTNR1B*)	g. 10C>T (*MTNR1C*)	g. 24A>G (*MTNR1C*)	g. 108C>T (*MTNR1C*)	g. 363T>C (*MTNR1C*)
g. 268C>T (*MTNR1A*)	-	0.04	0.04	0.07	0.10	0.02	0.05
g. 41C>T (*MTNR1B*)	0.30	-	1.00	0.21	0.33	0.32	0.22
g. 161T>C (*MTNR1B*)	0.30	1.00	-	0.21	0.33	0.32	0.22
g. 10C>T (*MTNR1C*)	0.56	0.04	0.04	-	0.49	0.63	0.71
g. 24A>G (*MTNR1C*)	0.56	0.08	0.08	0.18	-	0.22	0.75
g. 108C>T (*MTNR1C*)	0.54	0.03	0.03	0.13	0.91	-	0.46
g. 363T>C (*MTNR1C*)	0.55	0.03	0.03	0.36	0.29	0.03	-

*LD were performed for each SNP using the SHEsisPlus online software platform (http://shesisplus.bio-x.cn/SHEsis.html). The figure above diagonal line means the value of D' and the figure under diagonal line mean the value of r^2.

Table S2 Relationship between *MTNR1A* (g. 268C>T) genotype and color of eggshell in Shaoxing ducks

Genotype[1]	Number of white eggshell[2]	Number of blue eggshel1[2]	OR[3]	95%CI
TT	35 (4.46)	7 (0.89)	5.671	2.468-13.030
CT	180 (22.93)	102 (12.99)	2.002	1.477-2.713
CC	216 (27.52)	245 (31.21)	1.000	

[1]Genotype: number of white eggshell versus number of blue eggshell: χ^2=34.848, *P*=0.000 (3×2 contingency table). [2]Data reported as n (%). [3] Reference group (CC) designated with an OR of 1.000.
